# Role of Primary Healthcare Physicians in Early Detection of Colorectal Cancer in Al-Ahsa, Saudi Arabia

**DOI:** 10.7759/cureus.48732

**Published:** 2023-11-13

**Authors:** Mohammed Alessa, Fatema M Alhelal, Abdulelah A Al Ahmed, Abdullah A AlKhars, Ridha K Alomran, Mohammed M Albaqshi, Norah I Alabdullatif, Ziyad Alamer, Ali H Buzaid, Hussain Abbas

**Affiliations:** 1 General Surgery, King Faisal University, Al-Ahsa, SAU; 2 College of Medicine, King Faisal University, Al-Ahsa, SAU; 3 General Surgery, King Fahad General Hospital, Al-Ahsa, SAU; 4 General Surgery, King Fahad Hospital, Al-Ahsa, SAU

**Keywords:** early detection of cancer, primary health care centers, screening guidelines, primary healthcare physician, colorectal cancer

## Abstract

Introduction: Colorectal cancer (CRC) is the third most common cancer in Saudi Arabia. Late stages of the disease are associated with increased mortality rates, and early detection is known to improve the disease course and significantly reduce the mortality rate. Physicians’ knowledge and practices regarding CRC screening guidelines influence the successful implementation of screening programs. Understanding them is key to developing targeted interventions to enhance screening rates and promote early detection.

Methods: This study was a cross-sectional assessment of the current practice and knowledge of CRC screening among healthcare practitioners in Al-Ahsa, Saudi Arabia, by using a questionnaire. This questionnaire had seven multiple-choice questions to assess knowledge and six multiple-choice questions to assess physicians' attitudes toward CRC screening.

Results: The mean age of participants was 33 years; 60.8% (n=113) were men and 39.2% (n=73) were women. The majority were Saudi nationals (n=169; 90.9%). Self-assessed knowledge levels varied: 42.5% considered their knowledge of CRC screening adequate, 27.4% indicated that it was poor, and 30.1% reported that it was satisfactory. Positive attitudes towards CRC screening were expressed by 83.9% of participants. Also, physicians’ attitude scores varied by demographic factors. Significant correlations were found between attitude scores and marital status, medical qualification, and job title. There was no significant correlation between gender, nationality, and years of experience. The majority (75.3%) agreed that colonoscopy is the best available screening test, but highlighted issues with accessibility and actual availability.

Conclusion: Findings from this study provide insights into physicians’ knowledge, attitudes, and practices regarding CRC screening. Understanding these factors is crucial for developing effective interventions to enhance CRC screening rates and overall public health. Further education and standardized guidelines are recommended to address the variations observed in the study population.

## Introduction

Colorectal cancer (CRC) arises due to uncontrolled cell replication in the colonic mucosa [1]. Many different types of CRC have been discovered and are classified based on histopathological appearance [2]. Numerous risk factors contribute to the progression of CRC, including genetic predisposition, level of physical activity, nutritional pattern, obesity, smoking, and alcohol intake [3]. CRC has the third-highest incidence rate and the second-highest mortality rate worldwide [4]. It is most common in industrialized countries due to sedentary lifestyles [5]. CRC was the most common cancer in Saudi Arabia in 2020, accounting for 14.04% of all cancers [6]. Colonic and rectal cancers were respectively the third and sixth most common causes of death among individuals with cancer in Saudi Arabia in 2020 [6].

When CRC treatment begins at an early stage of the disease, the outcomes improve significantly, while worse prognoses are associated with the metastatic stages [7]. Screening programs can significantly improve disease outcomes by identifying cases at an early stage. CRC screening in Saudi Arabia should ideally begin at 45 years of age [8]. However, the average age of CRC detection in men and women has been reported to be 60 and 55 years, respectively [9]. A study with approximately 3,000 participants with an average age of 70 years found that the CRC screening rate was only 5.64% [10]. Many factors potentially contribute to the low rate of CRC screening, including lack of physician knowledge and practice. For example, one study found that only 55% of primary healthcare physicians in Saudi Arabia followed CRC screening guidelines [11]. In addition, there may also be low awareness of the importance of CRC screening [12]. The aim of this study was to assess whether primary healthcare physicians in Alhassa, Saudi Arabia, are following CRC screening guidelines.

## Materials and methods

This was a cross-sectional study conducted from March 2023 to May 2023 targeting physicians in primary health care centres (PHCs) in Al-Ahsa region. Data was collected by filling electronic questionnaire by interviewing the doctors. A total of 186 participants were included, calculated taking a 5% margin of error, and a 95% confidence interval. The study was approved by King Faisal University, Al-Ahsa, Saudi Arabia (approval number: KFU-REC-2022-DEC-ETHICS413).

The questionnaire used in this study was adapted from a validated questionnaire on the screening of colorectal carcinoma as described by Mosli et al. [11]. Seven multiple-choice questions were used to assess knowledge, and six multiple-choice questions were used to assess the attitudes of physicians toward CRC screening. Each accurate answer was scored as 1 point, whereas each wrong or “do not know” response received a score of 0. Bloom’s classification cutoff points for knowledge were applied: a knowledge score between 75% and 100% was regarded as adequate; a score between 50% and 74% was satisfactory; and a score of less than 50% was considered poor. A score greater than the mean was indicative of a positive attitude, while a score less than the mean indicated a negative attitude. Cronbach’s alpha was used to assess the internal consistency and reliability of a set of items within a questionnaire or scale.

The analysis included both descriptive and inferential statistical tests. Descriptive statistics were used to summarize and describe the characteristics of the study participants and the questionnaire findings. Frequencies and percentages were calculated for categorical variables. For continuous variables such as knowledge and attitude scores, the mean and standard deviation were calculated for normally distributed variables, and the median and interquartile range were used for non-normally distributed variables. The normality of the data was assessed using the Shapiro-Wilk test. Due to the non-normal distribution of the data, the independent-samples Kruskal-Wallis test was used to compare knowledge and attitude scores between different demographic groups. Fisher’s exact test was used to examine the association between demographic characteristics and CRC screening practice. The significance level for all statistical tests was set to P < 0.05, indicating a 95% confidence interval. All statistical calculations were performed using IBM SPSS Statistics for Windows, Version 27.0 (Released 2020; IBM Corp., Armonk, New York, United States).

## Results

Table 1 shows the demographics of the study participants. The mean age of participants was 33 years. The group was predominantly composed of men (n=113; 60.8%) and Saudi nationals (n=169; 90.9%). Women (n=73; 39.2%) and non-Saudi nationals (n=17; 9.1%)made up lower proportions. Most participants were married (n=131; 70.4%), while 49 (26.3%) were single and six (3.2%) were divorced. A large proportion of the participants held a Board-certified degree or PhD in Family Medicine (n=89; 47.8%) or a Bachelor of Medicine and Bachelor of Surgery (MBBS) degree (n=66; 35.5%). A smaller proportion held diplomas or master’s degrees in Family Medicine (n=8; 4.3%) or other specialties (n=2; 1.1%). A minority of participants possessed Board-certified or PhD qualifications in other specialties (n=21; 11.3%). With regard to experience, the majority of participants (n=99; 53.2%) reported having 3-10 years of experience, while 24.2% (n=45) had fewer than two years of experience and 22.6% (n=42) had more than 10 years of experience. The job titles of the participants included consultant (n=45; 24.2%), resident (n=96; 51.6%), and specialist (n=45; 24.2%).

**Table 1:** Demographic data of the study participants MBBS: Bachelor of Medicine and Bachelor of Surgery

		Mean	Standard Deviation
Age		33	6
		n	%
Gender	Female	73	39.2
	Male	113	60.8
Nationality	Non-Saudi	17	9.1
	Saudi	169	90.9
Marital status	Divorced	6	3.2
	Married	131	70.4
	Single	49	26.3
Medical qualification	Board or PhD in Family Medicine	89	47.8
	Board or PhD in other specialty	21	11.3
	Diploma or Master’s in Family Medicine	8	4.3
	Diploma or Master’s in other specialty	2	1.1
	MBBS	66	35.5
Years of experience	>10	42	22.6
	≤2	45	24.2
	3–10	99	53.2
Job title	Consultant	45	24.2
	Resident	96	51.6
	Specialist	45	24.2

Table 2 shows the participants’ knowledge and practices regarding CRC screening. When initiating screening for asymptomatic patients with average risk, 68.8% (n=128) of the participants recommended starting at the age of 50, 20.4% (n=38) suggested age 40, and 3.8% (n=7) proposed starting screening at age 60. When asked about an upper age limit for discontinuing screening, 63.0% (n=117) did not specify an age, 19.0% (n=35) recommended the age of 75, 11% (n=21) suggested below 75 years, and 7% (n=7) above 75 years.

**Table 2:** Knowledge of colorectal cancer screening FOBT: fecal occult blood test

Question	Response	n	%
For the majority of your asymptomatic patients with average risk, you will start screening at age of:	60 years	7	3.8
	40 years	38	20.4
	50 years	128	68.8
	Not sure	13	7.0
Is there an age at which you no longer recommend screening for healthy patients?	Yes, more than 75 years old	13	7
	Less than 75 years old	21	11
	75 years	35	19
	No	117	63
The frequency of screening with FOBT is every:	Not sure	42	22.6
	1 year	112	60.2
	3 years	15	8.1
	2 years	17	9.1
The frequency of screening with colonoscopy is every:	1 year	21	11.3
	10 years	91	48.9
	3 years	13	7.0
	5 years	32	17.2
The frequency of screening with sigmoidoscopy is every:	1 year	10	5.4
	10 years	11	5.9
	3 years	30	16.1
	5 years	98	52.7
	Not sure	37	19.9
For colorectal cancer screening using FOBT, how many samples should you order?	Not sure	35	18.8
	One	71	38.2
	Three	33	17.7
	Two	47	25.3

Participants reported using various screening practices. When asked about the use of the fecal occult blood test (FOBT), 60.2% (n=112) favored annual screening, 9.1% (n=17) and 8.1% (n=15) endorsed screenings every two and three years, respectively, and 22.6% (n=42) were uncertain. Regarding colonoscopy frequency, 48.9% (n=95) recommended it every 10 years, 17.2% (n=32) every five years, and 7.0% (n=13) chose three years. For sigmoidoscopy, 52.7% (n=98) preferred screening every five years, 16.1% (n=30) supported screening every three years, and 19.9% (n=37) were unsure. Regarding FOBT sample order numbers, 38.2% (n=71) suggested one, 25.3% (n=47) recommended two, 17.7% (n=33) preferred three, and 18.8% (n=35) were unsure.

Table 3 presents the participants’ perceptions of the effectiveness of various screening procedures in reducing CRC mortality among average-risk patients aged 50 years and older. When asked about overall effectiveness, an overwhelming majority (n=181; 97.3%) of participants expressed belief in its effectiveness, with only 2.7% (n=5) responding negatively.

**Table 3:** Attitudes toward colorectal cancer screening

Question	Response	n	%
Do you think that colorectal cancer screening for asymptomatic average-risk patients aged 50 years and older is effective?	No	5	2.7
	Yes	181	97.3
How effective do you believe the following screening procedures are in reducing colorectal cancer mortality in average-risk patients aged 50 years and older?			
Fecal occult blood testing	Do not know	11	5.9
	Effective	162	87.1
	Not effective	13	7.0
Flexible sigmoidoscopy	Do not know	21	11.3
	Effective	154	82.8
	Not effective	11	5.9
Colonoscopy	Do not know	5	2.7
	Effective	169	90.9
	Not effective	12	6.5
Double-contrast barium enema	Do not know	40	21.5
	Effective	101	54.3
	Not effective	45	24.2
CT colonography	Do not know	28	15.1
	Effective	128	68.8
	Not effective	30	16.1

Regarding FOBT, 87.1% (n=162) of participants considered it to be effective, 7% (n=13) viewed it as not effective, and 5.9% (n=11) were unsure. Similarly, the majority (n=154; 82.8%) believed flexible sigmoidoscopy to be effective, while 5.9% (n=11) considered it as not effective, and 11.3% (n=21) were unsure. A larger proportion (n=169; 90.9%) considered colonoscopy to be effective in reducing CRC mortality. Only 54.3% (n=101) considered double-contrast barium enema to be effective; 24.2% (n=54) considered it to be not effective, while 21.5% (n=40) expressed uncertainty. A majority (n=128;68.8%) believed computed tomography (CT) colonography to be effective, while 16.1% (n=30) considered it not effective, and 15.1% (n=28) were unsure.

Table 4 presents the practice patterns of study participants concerning CRC screening. The majority (n=155; 83.3%) reported performing CRC screening for asymptomatic patients with average risk aged 50 years and older. However, 16.7% (n=31) indicated that they did not order such screenings. A majority (n=104; 55.9%) reported ordering or performing the FOBT 1-10 times per month, 22% (n=41) ordered it 11-20 times, and 7% (n=13) ordered the FOBT 21-40 times. A small proportion (n=7;3.8%) ordered it more than 40 times per month, and 11.3% (n=21) did not order FOBT at all in a month. Sigmoidoscopy referral frequencies varied; 58.6% (n=109) of participants made no referrals, 19.4% (n=36) referred one to five times, 18.8% (n=35) referred 6-10 times, and 2.7% (n=5) referred 11-20 times per month. Regarding colonoscopy referrals in a month, 41.4% (n=77) of participants reported zero, 34.9% (n=65) reported one to five, 20.4% (n=38) reported 6-10, and 2.2% (n=4) reported 11-20. Participant preferences leaned slightly towards opportunistic screening (n=95; 51.1%) compared with structured screening programs (n=91; 48.9%). Reported initial follow-up steps following a positive FOBT varied. While 48% (n=90) recommended repeating the FOBT, 38% (n=71) suggested colonoscopy, 15% (n=28) suggested flexible sigmoidoscopy, 4% (n=8) suggested double contrast barium enema, and 2% (n=5) recommended CT colonography. Participant responses diverged regarding the next steps following a negative second FOBT; 46% (n=87) did not halt the work-up, 33.9% (n=63) did, and 19.4% (n=36) were unsure.

**Table 4:** Practice of colorectal cancer screening FOBT: fecal occult blood test

Question	Response	n	%
Do you perform colorectal cancer screening for asymptomatic average-risk patients aged 50 years and older?	No	31	16.7
	Yes	155	83.3
During a typical month, how many times do you order or perform the FOBT screening test?	1–10	104	55.9
	11–20	41	22.0
	21–40	13	7.0
	>40	7	3.8
	0	21	11.3
During a typical month, how many times do you refer asymptomatic average-risk patients for screening sigmoidoscopy?	0	109	58.6
	1–5	36	19.4
	11–20	5	2.7
	6–10	35	18.8
	>20	1	0.5
During a typical month, how many times do you refer asymptomatic average-risk patients for screening colonoscopy?	0	77	41.4
	1–5	65	34.9
	11–20	4	2.2
	6–10	38	20.4
	>20	2	1.1
To whom do you usually refer your patients for screening colonoscopy?	Gastroenterology	123	66.1
	Internal medicine	12	6.5
	Oncology	1	0.5
	Surgery	50	26.9
Which one of the following ways of conducting colorectal cancer screening do you prefer?	Opportunistic screening	95	51.1
	Structured screening program	91	48.9
Which of the following do you usually recommend to a healthy average-risk patient as an initial follow-up step to a positive FOBT?	Colonoscopy	71	38
	Double-contrast barium enema	8	4
	CT colonography	5	2
	Flexible sigmoidoscopy	28	15
	Repeat FOBT	90	48
Do you stop the work-up if the second FOBT is negative?	I don’t know	36	19.4
	No	87	46.8
	Yes	63	33.9

The screening test combinations that participants discuss with their patients are shown in Table 5. The majority (n=86;46.2%) discussed FOBT with their patients. One person (0.05%) mentioned FOBT without further details. A minority (n=23;12%) discussed colonoscopy as a screening test option. In addition, 11.3% (n=21) discussed the combination of FOBT, sigmoidoscopy, and colonoscopy.

**Table 5:** Screening test combinations discussed with patients

Screening tests		n	%
	Colonoscopy	23	12
	Fecal occult blood test	86	46.2
	Fecal occult blood test	1	0.05
	Fecal occult blood test, colonoscopy	26	13.9
	Fecal occult blood test, colonoscopy, fecal immunological test (FIT)	1	0.5
	Fecal occult blood test, colonoscopy for high-risk group	1	0.5
	Fecal occult blood test, FIT	5	2.0
	Fecal occult blood test, sigmoidoscopy	2	1.0
	Fecal occult blood test, sigmoidoscopy, colonoscopy	21	11.3
	Fecal occult blood test, sigmoidoscopy, colonoscopy, flexible sigmoidoscopy	2	1.0
	Fecal occult blood test, sigmoidoscopy, colonoscopy, possibly transferred for other investigations	1	0.5
	FIT	4	2.1
	Sigmoidoscopy	3	1.6
	Sigmoidoscopy, colonoscopy	9	4.8

A significant proportion of participants (n=64; 34.4%) indicated that their workplace does not have a policy or procedure in place for CRC screening (Table 6). Participants also reported variations in the presence of reminder systems for screening in their workplace; 73 (39.2%) indicated that reminders are usually available. Patient adherence presented challenges, with 54.3% (n=101) of participants reporting that patients sometimes do not follow through to complete CRC screening tests. The availability of trained providers of procedures was also an issue; 61.3% of participants indicated a shortage of providers of non-FOBT screening tests sometimes or usually, and 56.5% of participants reported a shortage of providers of procedures for follow-up on a positive screening test.

**Table 6:** Barriers to colorectal cancer screening for asymptomatic, average-risk patients

Barrier	Frequency	n	%
There is no policy and procedure in my workplace for screening.	Never	64	34.4
	Rarely	31	16.7
	Sometimes	49	26.3
	Usually	42	22.6
There is no reminder system in my workplace.	Never	31	16.7
	Rarely	23	12.4
	Sometimes	59	31.7
	Usually	73	39.2
Patients do not follow through to complete colorectal cancer screening tests.	Never	7	3.8
	Rarely	31	16.7
	Sometimes	101	54.3
	Usually	47	25.3
There is a shortage of trained providers to conduct screening other than FOBT.	Never	23	12.4
	Rarely	47	25.3
	Sometimes	67	36.0
	Usually	49	26.3
There is a shortage of trained providers to provide follow-up of positive screening tests with invasive endoscopic procedures.	Never	25	13.4
	Rarely	47	25.3
	Sometimes	61	32.8
	Usually	53	28.5

The published literature and guidelines influenced screening recommendations. A total of 143 participants (76.9%) indicated that clinical evidence in the published literature shapes their recommendations for CRC screening, while 75.8% (n=141) reported considering the United States Preventive Services Task Force recommendations and 76.3% (n=142) cited the American Cancer Society guidelines (Table 7). Locally, the availability of providers for referral beyond FOBT screening was deemed influential by 71.0% (n=132) of participants, and over half were influenced by the practice of their colleagues (n=114; 61.3%) or preferences of their patients (n=123; 66.1%). These results suggest that local practices and experiences contribute to shaping individual providers’ approaches to CRC screening.

**Table 7:** Factors that influence recommendations for colorectal cancer screening in practice

Factor	Importance	n	%
Clinical evidence in the published literature	Influential	143	76.9
	Not applicable or not familiar with	27	14.5
	Not influential	16	8.6
United States Preventive Services Task Force recommendations	Influential	141	75.8
	Not applicable or not familiar with	22	11.8
	Not influential	23	12.4
American Cancer Society guidelines	Influential	142	76.3
	Not applicable or not familiar with	29	15.6
	Not influential	15	8.1
Availability of providers to whom I can refer my patients for screening other than FOBT	Influential	132	71.0
	Not applicable or not familiar with	33	17.7
	Not influential	21	11.3
How colleagues in my practice or local community provide colorectal cancer screening for their patients	Influential	114	61.3
	Not applicable or not familiar with	37	19.9
	Not influential	35	18.8
My patients’ preferences for colorectal cancer screening	Influential	123	66.1
	Not applicable or not familiar with	30	16.1
	Not influential	33	17.7

When asked specifically about colonoscopy, 140 participants (75.3%) agreed that colonoscopy is the best available screening test, although only half agreed that it is readily available for their patients (Table 8). Some respondents (n=62;33.3%) disagreed that it is the best available screening test, potentially indicating issues with accessibility or availability of colonoscopy services. A majority (n=103; 55.4%) agreed with the statement that specialists able to perform colonoscopies are often too busy to conduct them for screening purposes.

**Table 8:** Statements regarding colonoscopy for colorectal cancer screening for asymptomatic, average-risk patients

Statement	Response	n	%
It is the best available screening test.	Agree	140	75.3%
	Disagree	26	14.0%
	Not applicable	20	10.8%
It is readily available for my patients.	Agree	93	50.0%
	Disagree	62	33.3%
	Not applicable	31	16.7%
The performing specialist is busy and cannot perform colonoscopy for screening purposes.	Agree	103	55.4%
	Disagree	43	23.1%
	Not applicable	40	21.5%

The median knowledge score was the same for both female (4) and male (4) physicians (Table 9). The interquartile range for both genders also showed similar patterns, with female physicians having a slightly larger range (4-6) than male physicians (5-6). Physicians of Saudi nationality had a significantly higher median knowledge score (4) compared with non-Saudi physicians (2). Knowledge scores were similar between physicians of different marital statuses. Those with a Board or PhD in Family Medicine qualification had the highest median knowledge score (5), followed by MBBS (3), Diploma or Master’s degree in Family Medicine (2), Diploma or Master’s degree in another specialty (1.5), and Board or PhD in another specialty (1). This difference was statistically significant between the groups (p-value=0.001). The reported number of years of experience was not significantly associated with the average knowledge score. However, consultants had the highest median knowledge score (5), followed by specialists (5), and residents (3). This difference was statistically significant (P = 0.001).

**Table 9:** Knowledge score compared by demographic characteristics MBBS: Bachelor of Medicine and Bachelor of Surgery

		Knowledge score			P-value
		Median	75th Percentile	25% Percentile	
Gender	Female	4.00	6.00	2.00	0.628
	Male	4.00	5.00	2.00	
Nationality	Non-Saudi	2.00	3.00	1.00	0.001
	Saudi	4.00	6.00	3.00	
Marital status	Divorced	3.50	6.00	3.00	0.960
	Married	4.00	5.00	2.00	
	Single	4.00	5.00	2.00	
Medical qualification	Board or PhD in Family Medicine	5.00	6.00	4.00	0.001
	Board or PhD in another specialty	1.00	3.00	1.00	
	Diploma or master’s in Family Medicine	2.00	3.50	2.00	
	Diploma or master’s in another specialty	1.50	2.00	1.00	
	MBBS	3.00	5.00	2.00	
Years of experience	3–10 years	4.00	6.00	2.00	0.202
	≤2	4.00	5.00	2.00	
	>10 years	3.00	5.00	2.00	
Job title	Consultant	5.00	6.00	4.00	0.001
	Resident	3.00	4.00	2.00	
	Specialist	5.00	6.00	3.00	

Among the participants, 42.5% (n=79) had adequate knowledge of CRC screening (Table 10), and similar proportions had satisfactory or poor knowledge.

**Table 10:** Level of knowledge of colorectal cancer screening among physicians

Level of knowledge	n	%
Adequate	79	42.5
Poor	51	27.4
Satisfactory	56	30.1

The median attitude score was the same for both female (5) and male (5) physicians (Table 11). The interquartile range for both genders was also the same, suggesting that gender did not significantly influence participants’ attitudes toward CRC screening. Attitude scores were not significantly different between Saudi (5) and non-Saudi (6) physicians, but they did differ by marital status. Divorced physicians had a median attitude score of 4.50, married physicians had a higher median score of 6, and single physicians had a median score of 5. This difference was statistically significant (P = 0.029). Physicians with a Board or PhD in Family Medicine or Board or PhD in another specialty had the highest median attitude score (6), followed by MBBS (5), Diploma or Master’s degree in Family Medicine (4), and Diploma or Master’s degree in another specialty (3.5). This difference was statistically significant (P = 0.001), suggesting that specialized qualifications in Family Medicine were associated with more positive attitudes. Years of experience did not appear to have a significant impact on attitudes (P = 0.420), and the median attitude scores were consistent across different experience levels. However, significant differences were observed based on job titles. Consultants and specialists had the highest median attitude score (6), followed by residents (5). This difference was statistically significant (P = 0.003).

**Table 11:** Attitude scores by demographic characteristics

		Attitude score			P-value
		Median	75th Percentile	25th Percentile	
Gender	Female	5.00	6.00	4.00	0.927
	Male	5.00	6.00	4.00	
Nationality	Non-Saudi	6.00	6.00	4.00	0.588
	Saudi	5.00	6.00	4.00	
Marital status	Divorced	4.50	5.00	3.00	0.029
	Married	6.00	6.00	4.00	
	Single	5.00	6.00	4.00	
Medical qualification	Board or PhD in Family Medicine	6.00	6.00	4.00	0.001
	Board or PhD in another specialty	6.00	6.00	5.00	
	Diploma or master’s in Family Medicine	4.00	5.50	2.00	
	Diploma or master’s in another specialty	3.50	4.00	3.00	
	MBBS	5.00	6.00	4.00	
Years of experience	3–10	5.00	6.00	4.00	0.420
	≤2	5.00	6.00	4.00	
	>10	5.50	6.00	4.00	
Job title	Consultant	6.00	6.00	4.00	0.003
	Resident	5.00	6.00	4.00	
	Specialist	6.00	6.00	5.00	

Most participants (156; 83.9%) expressed a positive attitude toward CRC screening (Table 12).

**Table 12:** Attitudes toward colorectal cancer screening

Attitude	n	%
Negative	30	16.1
Positive	156	83.9

Chi-squared testing showed a significant association between nationality and CRC screening practice (χ2(1) = 4.674, P = 0.031)) and medical qualification and CRC screening (χ2(4) =25.050, P < 0.001) (Table 13). Job title was also significantly associated with CRC screening practices(χ2(2) = 7.580, P = 0.023).

**Table 13:** Colorectal cancer screening practices by demographic characteristics MBBS: Bachelor of Medicine and Bachelor of Surgery

		Do you perform colorectal cancer screening for asymptomatic average-risk patients aged 50 years and older?				P-value
		No		Yes		
		n	%	n	%	
(2) Gender	Female	14	45.2	59	38.1	0.460
	Male	17	54.8	96	61.9	
(3) Nationality	Non-Saudi	6	19.4	11	7.1	0.031
	Saudi	25	80.6	144	92.9	
(4) Marital status	Divorced	1	3.2	5	3.2	0.997
	Married	22	71.0	109	70.3	
	Single	8	25.8	41	26.5	
(6) Medical qualification	Board or PhD in Family Medicine	7	22.6	82	52.9	0.001
	Board or PhD in another specialty	11	35.5	10	6.5	
	Diploma or Master’s degree in Family Medicine	2	6.5	6	3.9	
	Diploma or Master’s degree in another specialty	0	0.0	2	1.3	
	MBBS	11	35.5	55	35.5	
(7) Years of experience	3–10	15	48.4	84	54.2	0.513
	≤2	10	32.3	35	22.6	
	>10	6	19.4	36	23.2	
(8) Job title	Consultant	2	6.5	43	27.7	0.023
	Resident	22	71.0	74	47.7	
	Specialist	7	22.6	38	24.5	

There was no significant difference in screening practices between physicians who reported being influenced by clinical evidence in published literature or guidelines, and those who did not (Table 14). However, a significant difference was found between those who reported being influenced by the availability of providers, their colleagues’ practice, or patient preferences, and those who did not.

**Table 14:** Colorectal cancer screening practice influences by different guidelines FOBT: fecal occult blood test

	Perform Colorectal Cancer Screening				P-value
	No		Yes		
	n	%	n	%	
Clinical evidence in the published literature	20	14.0	123	86.0	0.201
United States Preventive Services Task Force recommendations	19	13.5	122	86.5	0.093
American Cancer Society guidelines	22	15.5	120	84.5	0.741
Availability of providers to whom I can refer my patients for screening other than FOBT	16	12.1	116	87.9	0.004
How colleagues in my practice or local community provide colorectal cancer screening for their patients	11	9.6	103	90.4	0.001
My patients’ preferences for colorectal cancer screening	10	8.1	113	91.9	0.001

## Discussion

CRC is a significant global health concern, ranking among the most common and deadly cancers. Early detection and effective screening programs play a vital role in reducing CRC-related morbidity and mortality. A recent study found that CRC screening prevalence is very low [10]. However, physicians can be pivotal in guiding patients toward appropriate screening methods and recommendations. This study aimed to explore the knowledge and practices of family medicine physicians in Saudi Arabia regarding CRC screening. The results shed light on current practices and identify areas for improvement [8].

The study’s participants included physicians with diverse demographic characteristics. The mean age of the participants was 33 years and the gender distribution was 60.8% (n=113) male and 39.2% (n=73) female. A majority (n=169; 90.9%) of participants were Saudi nationals, with the rest being non-Saudi (n=17; 9.1%). Marital status varied, with 70.4% (n=131) married, 26.3% (n=49) single, and 3.2% (n=6) divorced participants. They held a variety of medical qualifications: 47.8% (n=89) had a Board or Ph.D. in Family Medicine, and 35.5% (n=66) had an MBBS degree. Years of experience also varied, and a majority (n=99; 53.2%) had 3-10 years of experience. Job titles included consultant (n=45; 24.2%), resident (n=96;51.6%), and specialist (n=45; 24.2%). Similar demographic characteristics were found in another study [11].

Our study found diverse CRC screening knowledge and practice patterns. Most participants (n=128; 68.8%) recommended initiating CRC screening at age 50, and 20.4% (n=38) suggested age 40. Surprisingly, 3.8% (n=7) proposed initiating screening at age 60. Another study reported that 64% of participants believed that most asymptomatic, average-risk patients should commence screening at the age of 45 years [11]. There were discrepancies in recommendations for screening frequencies in the current study, such as 60.2% (n=112) favoring annual FOBT screening and 48.9% (n=91) suggesting colonoscopy every 10 years. Similarly, variations emerged in opinions on the age at which screening should start and stop. These findings are similar to those of a study conducted in Riyadh [11].

Physicians displayed predominantly positive attitudes towards CRC screening. Almost all believed CRC screening for asymptomatic average-risk patients aged 50 years and above to be effective. Positive attitudes were observed toward various screening methods including FOBT, flexible sigmoidoscopy, colonoscopy, double-contrast barium enema, and CT colonography. These attitudes were similar to the findings of a study by Alghamdi et al., which also showed that a majority of participants had a positive attitude toward CRC screening [13].

Our study identified barriers affecting CRC screening practices. Some physicians reported a lack of policies and procedures for screening (n=64; 34.4%) and a shortage of trained providers of non-FOBT methods (n=116; 62.3%). Patient adherence issues were reported by 54.3% (n=101) of participants. Factors influencing screening decision-making included clinical evidence in published literature, the United States Preventive Services Task Force recommendations, the American Cancer Society guidelines, availability of investigation providers, colleagues’ practices, and patient preferences. This is in contrast to the findings of a study conducted in Malaysia, where the most common barrier stated by the primary care providers was “unavailability of the test” [14]. The two most common patient factors were “patient in a hurry” and “poor patient awareness.”

Certain demographic characteristics were found to be related to participants’ knowledge and attitudes. Saudi nationality, specialized medical qualifications in family medicine, and the job titles of consultant and specialist were associated with higher knowledge scores. These results were comparable to another study [11]. Marital status (being married) and specialized medical qualifications in family medicine were linked to more positive attitudes towards CRC screening in our findings. However, no association was found in the study by Mosli et al. [11].

Nationality, medical qualification, and job title were significantly associated with CRC screening practices among healthcare professionals. Tailoring interventions based on these demographic factors could enhance CRC screening rates and early cancer detection. The findings demonstrated statistically significant associations between recommendations for CRC screening and factors such as the availability of providers (P = 0.004), colleague practices (P = 0.001), and patient preferences (P < 0.001). However, other factors such as clinical evidence and guidelines did not significantly influence recommendations. This finding is in contrast to another study that reported that physicians whose practice was influenced by the United States Preventive Services Task Force and American Cancer Society recommendations reported practicing CRC screening more than those who were not influenced by these resources [15].

The findings of this study have significant implications for enhancing CRC screening practices. Physicians’ knowledge gaps and variable practices highlight the importance of continuous medical education and standardized guidelines [14]. Efforts to improve patient adherence, streamline screening policies, and address provider shortages are crucial for the implementation of effective screening. Moreover, understanding the factors that influence physicians’ attitudes can aid in tailoring interventions to promote more positive attitudes toward CRC screening [16]. This study contributes to the ongoing efforts to reduce morbidity and mortality associated with CRC through informed and effective screening practices.

There were some limitations to our study. The participants were from a specific geographical location and may not be representative of physicians beyond this area. Additionally, the study relied on self-reported data, which can introduce bias. Future research could include the effectiveness of targeted interventions to address the knowledge gaps and barriers identified in this study. Comparative studies across different healthcare systems and regions could provide a more comprehensive understanding of the challenges and opportunities in CRC screening practices among physicians.

## Conclusions

Our research sheds light on CRC screening knowledge, attitudes, and practices among physicians. Demographics such as nationality, qualification, and job title were linked to varying levels of knowledge and attitudes regarding CRC screening. Saudi physicians and those specializing in Family Medicine displayed higher CRC screening knowledge. Married physicians also held more positive attitudes. Job title and medical qualification played a role, with consultants and specialists indicating better understanding and attitudes. Most participants viewed CRC screening positively, although a notable number showed negative attitudes, indicating a need for targeted education. Factors affecting screening practices included referral availability, colleagues’ practices, and patient preferences, which represent local and patient-centric influences. The study underscores the importance of tailored interventions to enhance CRC screening rates and ultimately improve patient outcomes.
